# Mesenchymal stem cell therapy for non-healing diabetic foot ulcer infection: New insight

**DOI:** 10.3389/fbioe.2023.1158484

**Published:** 2023-04-13

**Authors:** Golnaz Mahmoudvand, Arian Karimi Rouzbahani, Zahra Sadat Razavi, Mohamad Mahjoor, Hamed Afkhami

**Affiliations:** ^1^ Student Research Committee, USERN Office, Lorestan University of Medical Sciences, Khorramabad, Iran; ^2^ Physiology Research Center, Faculty of Medicine, Iran University of Medical Sciences, Tehran, Iran; ^3^ Department of Immunology, Faculty of Medicine, Iran University of Medical Sciences, Tehran, Iran; ^4^ Nervous System Stem Cells Research Center, Semnan University of Medical Sciences, Semnan, Iran; ^5^ Department of Medical Microbiology, Faculty of Medicine, Shahed University, Tehran, Iran

**Keywords:** diabetic foot, foot ulcer, wound healing, mesenchymal stem cell, multipotent stem cell

## Abstract

Diabetic foot ulcer (DFU) is considered the most catastrophic complication of diabetes mellitus (DM), leading to repeated hospitalizations, infection, gangrene, and finally amputation of the limb. In patients suffering from diabetes mellitus, the wound-healing process is impaired due to various factors such as endothelial dysfunction and synthesis of advanced glycation end-products, hence, conventional therapeutic interventions might not be effective. With increasing therapeutic applications of mesenchymal stem cells (MSCs) in recent years, their potential as a method for improving the wound-healing process has gained remarkable attention. In this field, mesenchymal stem cells exert their beneficial effects through immunomodulation, differentiation into the essential cells at the site of ulcers, and promoting angiogenesis, among others. In this article, we review cellular and molecular pathways through which mesenchymal stem cell therapy reinforces the healing process in non-healing Diabetic foot ulcers.

## Graphical Abstract

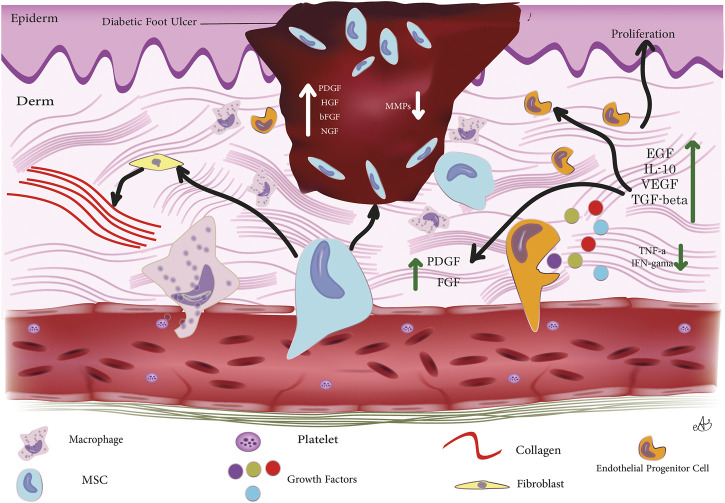

## 1 Introduction

Any type of damages or injuries on the surface of the skin are called skin wounds. Wounds can be caused by accidents such as burns, cuts, skin tears, or surgery. In addition, as a result of some diseases, a ground is provided for the creation of wounds or skin problems, such as diabetes mellitus (DM) that may cause wounds or diseases such as eczema or psoriasis (Bian et al., 2022). A diabetic foot ulcer (DFU) is one of the significant complications of DM and constitutes the largest proportion of diabetic foot complications. About 15% of people with DM suffer from DFU, and about 84% of cases of amputation of the lower part of the foot (Han et al., 2022). In the last 20 years, the increase in mortality rate of diabetic patients has been primarily due to the vascular complications of DM, which include involvement of small vessels such as kidney involvement and retinopathy, and involvement of large vessels including the vessels of the end of the lower limbs, which is followed by disruption of the healing process (Rehak et al., 2022). Immune cells play an important role in the difficult-to-heal pathological process of diabetic wounds (DWs) (Xiao et al., 2021). Constant inflammatory factor release from neutrophil infiltration, macrophage imbalance, mast cell degranulation, and dendritic cell and T cell dysregulation in DW causes inflammatory cascades, vascular maturation disorders, and decreased collagen deposition, all of which contribute to DW’s protracted or non-healing course (Pomatto et al., 2021). Mesenchymal stem cells (MSCs) have the ability to develop into a wide variety of cell types, including bone cells (osteoblasts), cartilage cells (chondrocytes), muscle cells (myocytes), and adipose tissue (adipocytes) (Wu et al., 2007). Researchers found that normal and DWs wound healing was much accelerated by the injection of GFP + allogeneic BM-MSCs surrounding the wound and their application to the wound bed using an excisional wound splinting paradigm (Wang et al., 2022).

## 2 Pathogenesis and characteristics of the diabetic foot ulcers

The burden of DM has significantly risen over recent years and DM is now regarded as an impending health emergency worldwide (Spiliopoulos et al., 2021). DFU is a common complication of DM, leading to significant morbidity and mortality (Mariadoss et al., 2022). DM is the leading cause of lower extremity amputation, causing more than one million limb losses among patients with DM annually (Spiliopoulos et al., 2021). DFU has been known as the most catastrophic complication of DM owing to repeated and long-term hospitalizations, various infections, and gangrene of the limb (Yu et al., 2022a). DFU is commonly graded from 0 (no lesion) to 5 (severe foot damage) by physicians (Mariadoss et al., 2022). The pathogenesis of DFU is multifactorial and is in close collaboration with poor glycemic control, peripheral vascular diseases, peripheral neuropathy, insufficient wound angiogenesis, improper foot care, and foot trauma (Alizadeh et al., 2019). Uncontrolled blood glucose levels are widely considered the primary factor in the processes of foot soft tissue disorder (Yu et al., 2022a). Published articles have reported that intensive diabetes management can diminish the likelihood of different conditions correlated with DFU including neuropathy. Moreover, the administration of insulin for diabetic patients has been described as a risk factor for the development of DFU. The possible reason for this finding is that this may indicate the severity of DM. Researchers have also demonstrated a relationship between high body mass index and an elevated chance of DFU (Al-Mohaithef et al., 2022). Other studies have also shown that the leading cause of DFU is the inability of modifying diabetic behavior among patients with low income. Hence, subjects with inadequate hygiene, a history of smoking, and alcohol consumption are estimated to be at a 20% chance of suffering from DFU (Mariadoss et al., 2022). Patients with DM are also more susceptible to developing peripheral arterial disease (PAD) compared with the general population. In these patients, PAD can be left undetected before tissue loss, since affected individuals may not encounter preceding manifestations like rest pain and claudication (Spiliopoulos et al., 2021). Furthermore, various growth factors have been found to play a role in the development of DM and its complications. Vascular endothelial growth factor (VEGF) is among these factors (Li et al., 2018a). Diabetic patients with DFU have demonstrated remarkably higher levels of advanced oxidation protein products, malondialdehyde (MDA), and tumor necrosis factor-α (TNF-α) that are essential for soluble vascular endothelial growth factor receptor-1 secretion and subsequent reduction of VEGF (Abd El-Khalik et al., 2020). Different factors may interfere with the wound healing process in diabetic patients. High blood glucose levels result in the synthesis of advanced glycation end-products, and the elevated levels of these products disturb the process of wound healing (Yu et al., 2022a). Hyperglycemia and uncontrolled DM have also been correlated with a higher risk of complications, mortality, and the need for re-amputation in diabetic patients undergoing limb amputation due to DFU (Nayak and Kirketerp-Møller, 2016). Because of endothelial dysfunction, vasodilation is diminished in DM. Subsequently, intravascular pressure rises leading to the migration of inflammatory cells. Inflammatory cells release lytic enzymes which contribute to the impairment of healing. The weakened activity of the white blood cells can also damage the healing and management of DFUs. Moreover, the ankle joint equinus is linked to reduced venous blood flow of the lower limb and negatively affects wound healing. All of these factors create a vicious cycle that must be broken by appropriate strategies (Primadhi and Herman, 2021). DFU may be complicated by a broad variety of microorganisms leading to diabetic foot infection (DFI). Aerobic and Gram-positive microorganisms, in particular *Staphylococcus aureus*, are the most common causes of DFI. Among Gram-negative bacteria, *Escherichia coli* and *Pseudomonas aeruginosa* are the most identified microorganisms (Rubitschung et al., 2021). About 50%–80% of DFIs are considered unavoidable. Nevertheless, sufficient foot care, blood glucose level control, and attending routine check-up sessions by diabetic patients can be beneficial in preventing infections. Furthermore, diabetic subjects are more likely to develop DFU if they work in an environment with high exposure to infection. Thus, the occupation of patients may also be an important factor in the occurrence of DFU (Mariadoss et al., 2022).

## 3 Prefaced of stem cells function in wound repair process

SCs are described as undifferentiated cells that are able to proliferate and transform into various types of mature cells that form different tissues and organs in the body (Bahmad et al., 2021; Mahjoor et al., 2022). SCs are found in embryos and adult cells, and with each step of specialization, their differentiation potency decreases (Zakrzewski et al., 2019; Mahjoor et al., 2021). SCs have shown promising potential for cell therapies against unyielding diseases such as spinal cord injury, cancer immunotherapy, heart failure, Parkinson’s disease, and type 1 DM (Yamanaka, 2020). Several SCs have been discovered in mammalian tissues, including hematopoietic SCs, germline SCs, epithelial SCs, neural SCs, muscle SCs, and MSCs (Wang, 2019). MSCs are pluripotent SCs originated from mesoderm during the early stages of development (Wang, 2019; Wang et al., 2021). MSCs can be extracted from different tissues including bone marrow, adipose tissues, placenta, and umbilical cord (Naji et al., 2019). These cells have the ability to differentiate into various cells such as neuron-like cells, adipocytes, muscle cells, and osteoblasts, as a result, they are of great importance in cell therapy as well as tissue repair (Wang et al., 2021). In particular, the effects of MSC-exosomes on wound healing have gained remarkable attention in recent years. Cutaneous wound healing is an intricate process comprising the homeostasis, inflammatory, proliferative, and remodeling phases (Saberiyan et al., 2020; Bray et al., 2021). Exosomes derived from MSCs have been shown to improve wound healing. Exosomes are described as the smallest vesicles that are risen from endosomes. They can be derived from the extracellular microenvironment of various cells including SCs. They exert their healing effects by playing a role in almost all stages of the wound healing process (Yang et al., 2020a; Vu et al., 2021). MSCs transfer into injured areas and convert into native components of the injured areas. They secrete, cytokines, chemokines, and growth factors that facilitate tissue regeneration (Fu et al., 2019). MSC transplantation induces M2 polarization of macrophages and improves the wound healing process (He et al., 2019). The immunomodulation capacity of exosomes is supposed to be as same as that of MSCs. However, while MSCs regulate immune cells through cytokine synthesis, exosomes play their role through micro ribonucleic acid (miRNA). SC-originated exosomes can influence the proliferation stage of the wound healing process by inciting resident cell differentiation and proliferation, along with enhancing angiogenesis in damaged areas (Vu et al., 2021). The application of extracellular vesicles from adipose SCs and bone marrow-MSCs has resulted in complete re-epithelialization of wounds in a rabbit (Pelizzo et al., 2018). Similarly, Zhang et al. (2015) showed that transplanting human-induced pluripotent SC-derived MSCs to injury sites in a rat model led to the improvement of re-epithelialization, decrease in scar widths, elevation of collagen maturity, and promotion of angiogenesis. Shabbir et al. (2015)) investigated the role of MSC-derived exosomes in wound healing. They isolated human MSCs (hMSCs) from normal donor bone marrow and fibroblasts from the wound edge of diabetic patients. The results showed that the exosomes led to the improvement of proliferation and migration of fibroblasts isolated from normal donors and diabetic patients. Furthermore, MSCs have shown strong antimicrobial properties through direct and indirect mechanisms (Alcayaga-Miranda et al., 2017). Sutton et al. (2016) observed that hMSCs secreted molecules that showed antimicrobial effects *in vitro* against *P. aeruginosa*, *Streptococcus pneumonia*, and *S. aureus*, and influenced the rate of bacterial growth. Sung et al. (2016) investigated the *in vivo* antimicrobial effects of MSCs. They found that transplantation of MSC in mice caused *in vivo* downregulation of the inflammatory response and promoted bacterial clearance, resulting in elevated protection against *E. coli*-induced pneumonia. However, it should be noted that SC therapy has some limitations. This method is costly and slow, furthermore, it is challenging in terms of ethics and law. Hence, researchers still try to make improvements in the field of wound treatment (Vu et al., 2021). For example, novel sources of MSCs such as medical waste material and Wharton’s jelly, and new delivery systems such as three-dimensional collagen allograft have demonstrated great efficacy compared to traditional methods (Lee et al., 2016).

## 4 Cellular and molecular mechanisms in diabetic foot ulcer repairing

### 4.1 Molecular mechanisms

#### 4.1.1 Diabetic foot repair: The role of angiogenesis

It is currently possible to use MSCs to treat delayed or disrupted wounds, and several studies have demonstrated that MSCs can treat DW caused by diabetic complications. The most effective results have been obtained with muscle grafts (Kim et al., 2019). A favorable scar microenvironment can be created for wound healing by mobilizing MSCs, and DFUs can be strengthened by mobilizing MSCs. There are a number of factors that affect the healing process of a wound. Regeneration requires interactions between dermal and epidermal cells, as well as interactions between cells and cytokines; it has been proven that umbilical cord (UC)-MSCs enhance the levels of VEGF, basic fibroblast growth factor (bFGF), and hepatocyte growth factor (HGF) in DFUs when they are administered with them. A downstream route of phosphoinositide 3-kinases (PI3K), HGF, affects the production of VEGF *via* the c-MET signaling cascade.

hMSCs can secrete VEGF and HGF by activating a signaling pathway involving p38 mitogen-activated protein kinases (MAPKs). TNF-α is a component of the injured area that is necessary for the production of VEGF and HGF secretion. Growth factors and cytokines modulate cell behavior (including immune cells). These molecules are essential for cellular interactions (e.g., fibroblasts, keratin-forming cells, immune cells, and endothelial cells) (Kopan and Ilagan, 2009).

It has been demonstrated that VEGF should spatially upregulate platelet-derived growth factor (PDGF)-B and fibroblast growth factor 2 (FGF-2) in lesions, and also mobilize and attract bone marrow-derived cells in a structured way (Sprinzak and Blacklow, 2021). The BM houses two major types of SCs: hematopoietic SCs (HSCs) and MSCs. Incorporating endothelial progenitor cells (EPCs) and adult bone marrow-derived hematopoietic SCs (BM-HSCs), which are precursors to erythrocytes, platelets, and white blood cells, into the local microenvironment of the wound website has been shown to boost the healing process and tissue regeneration (Yang et al., 2020b). Inducing the production of angiogenic elements like VEGF, and MSCs can enhance DFU (Zhao et al., 2020). Critical to the healing process, neovascularization is impeded by hyperglycemic conditions, as shown by the declined levels of the endothelial cell marker CD31 at the wound sites (Chen et al., 2020). The positive role of MSCs in improving diabetic wound healing have been shown in several para-clinical and clinical studies. O’Loughlin and her colleagues showed that topical use of allogeneic MSCs bedded in a collagen scaffold propagated wound healing and raised angiogenesis in a rabbit model of diabetic ulcer. They reported a marked difference in wound closure when 1 × 10^6^ cells were administered (O’Loughlin et al., 2013). Since mi-RNA-205-5p is an immediate regulator of VEGF protein translation and is expressed in hMSCs, it was discovered that mi-RNA-205-5p should mainly target the 3′-untranslated region (UTR) of VEGF mRNA to suppress the translation of VEGF protein, thereby slowing the healing of diabetic foot wounds (Rehak et al., 2022). The endogenous RNA MALAT1 competes with mi-RNA-205-5p and is involved in metastasis-associated lung cancer. Patients tissue samples with DFU show the downregulation of MALAT1 expression (Jayasuriya et al., 2020). Overexpression of MALAT1 in MSCs reduced significantly levels of miRNA-205-5p, which in turn increased VEGF production and accelerated the development of endothelial mobile tubes in the DFU model developed by immune-deficient NOD/SCID mice. Reducing VEGF mRNA levels by overexpressing MALAT1 or eliminating mi-RNA-205-5p had no major effect (Zhu et al., 2019). However, MSCs benefit greatly from VEGF in the protein stage, when MALAT1 acts as a posttranscriptional promoter to increase VEGF protein expression. Silencing MALAT1 in diabetic mouse skin decreases Collagen I and Collagen III expression, which in turn reduces collagen deposition at sites of injury and slows recovery (Liu et al., 2019). Fibroblasts have a substantial role in the normal wound healing process by secreting collagens. Studies have shown that MALAT1 aids in ulcer healing by either stimulating fibroblasts to produce more collagen or by increasing VEGF production. Diabetes is associated with a reduction in the amount of nuclear factor erythroid 2-related factor 2 (Nrf2) in the blood (Anguiano-Hernandez et al., 2019).

**FIGURE 1:**
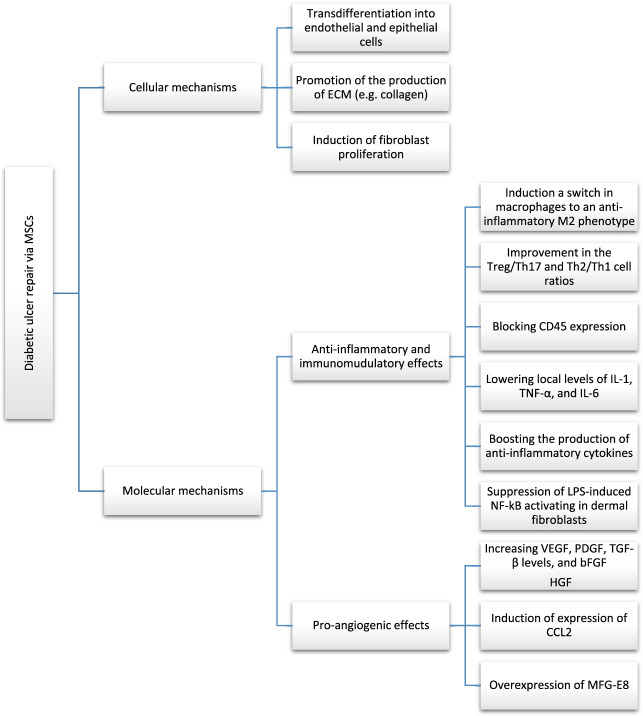
Cellular and molecular mechanisms through which MSCs are involved in diabetic foot ulcer repairing.

Through a positive feedback mechanism, Nrf2 may increase MALAT1 expression in DUF. Adipose-derived stromal cells (ADSCs) that overexpress Nrf2, release exosomes that aid in the healing process of DFUs. These exosomes promote cutaneous wound healing *via* boosting angiogenesis and EPC proliferation at the wound site. These findings provide credence to Nrf2’s critical function in regulating MSC-based treatments’ responses to an adverse *in vivo* microenvironment (Li et al., 2018b). In addition, elevating wound-local MALAT1 levels may greatly reduce inflammation and speed healing. Overexpressing MALAT1 in MSCs, for instance, has been shown to induce M2 macrophage polarisation and suppress pro-inflammatory cytokine IL-6 and TNF-α expression (Kuai et al., 2022). Heme oxygenase-1 (HO-1) is an Nrf2-dependent gene that plays a crucial function in the control of angiogenesis and the upregulation of angiogenic factors. Upregulation of HO-1 in BMSCs in STZ-induced diabetic mice has been shown to increase BMSC proliferation and VEGF production through the Akt signal pathway, which in turn greatly promotes wound ulcer healing (Hu et al., 2007). Essential for VEGF-induced AKT phosphorylation is the milk fat globule-epidermal growth factor 8 (MFG-E8), which is produced perivascularly and intravascularly. Important growth factors for inducing angiogenesis, such as VEGF and PDGF (Motegi et al., 2011), need to be signaled by MFG-E8 (Zhang et al., 2020). Overexpression of MFG-E8 by BM-MSCs has been shown to stimulate blood vessel formation. The underlying mechanism of MFG-E8 controlling inflammation is unknown, despite the fact that this research reveals that MFG-E8 may boost M2 macrophage infiltration and reduce pro-inflammatory cytokines, therefore facilitating DW repair (Uchiyama et al., 2017). The PI3K/Akt signaling pathway is essential for angiogenic progression (Liang et al., 2007). Prolonged expression of MSCs by binding to and activating the type 3 neurotrophin tyrosine kinase receptor (TrkC), NT-3-activated hMSC has been shown to speed the recovery of diabetic mice with foot wounds. According to some studies, NT-3 stimulates MSC proliferation *via* the Akt pathway. The release of VEGF and nerve growth factor (Ju et al., 2022) from MSCs may be stimulated by NT-3. In order to promote angiogenesis, these growth factors may stimulate Akt activation in vascular endothelial cells. Sub-culturing MSCs resulted in a gradual reduction in their c-jun expression. Also, downregulation of c-jun expression in DW locations was observed. Yue et al. (2020) discovered that boosting PDGFA and HGF levels with the local subcutaneous injection of hUC-MSCs overexpressing c-jun at the DW locations sped up angiogenesis and re-epithelialization. A number of cell types of mesenchymal origin, including smooth muscle cells and fibroblasts, rely on PDGFs as their primary mitogens. Activator protein-1 (AP1) is a major MAPK downstream effector, with critical roles in cell proliferation because of its primary component c-Jun. The ability of MSCs to express c-jun is critical for facilitating the healing of diabetic foot wounds. Increased c-jun expression in MSCs has been linked to a reduction in the production of matrix metalloproteinases 2 (MMP-2) and 9 (MMP-9), two enzymes that are detrimental to tissue healing (MMP-9). A vast body of evidence suggests the overexpression of MMP-2 and MMP-9 in chronic diabetic ulcers (Lin et al., 2022). The transcription of MMP-2 and MMP-9 has been demonstrated to be primarily regulated by AP-1 under a variety of circumstances, including DW. In contrast to the control group, DWs treated with bone marrow MSCs demonstrated a declining tendency in their expression of MMP-2 and MMP-9 after local application of MSCs. MMP-2 is crucial for sustained matrix remodeling and angiogenesis during wound healing, while MMP-9 may play a role in the remodeling of granulation tissue and the migration of keratinocytes (Wang and Gao, 2018). Furthermore, there are data suggesting that the suppression of MMP2 and MMP-9 *via* c-jun promotes wound healing in diabetic mice. It has been shown that elevated levels of MMP-2 and MMP-9 inhibit the breakdown of extracellular matrix (ECM) in the skin, which slows healing (Dalirfardouei et al., 2019). Figure 1 summarizes cellular and molecular mechanisms through which MSCs contribute to DFU repairing.

#### 4.1.2 Repairing diabetic foot through anti-inflammatory and immunomodulation

Low oxygen levels and an aberrant release of angiogenic signals are two potential outcomes of chronic inflammation (Ma et al., 2020). It has been hypothesised that the reparative effects of SC-based treatments are due, in part, to the anti-inflammatory and immunomodulatory effects of implanted SCs. The immune system is under the control of inflammation. Healing wounds benefit from low-grade inflammation because it aids in the elimination of invading pathogens, speeds the recovery of damaged tissues, and keeps the body in homeostasis (Arabpour et al., 2021). Delayed or non-healing DFUs are caused by persistent and severe inflammation. Immune suppression and redundant inflammation often coexist. Extreme immune reactions may result in serious systemic inflammation or allergic conditions, while weak immune reactions could quickly lead to serious or recurrent infections. To achieve tissue homeostasis after tissue damage, pro-inflammatory and anti-inflammatory cytokines must be in harmony at different phases of healing. Loretelli et al. (2020) discovered that a reduction in CD45^+^ inflammatory cells and interferon- (IFN-α) and an increase in regulatory T cells (Tregs), proliferating Ki-67+ cells, and the endothelial cell marker CD31 were observed following topical application of embryonic SC extracts in diabetic foot mice to boost wound healing of diabetic foot. These results demonstrate that embryonic SCs (ESCs) may regulate the immune response to aid in the repair of DWs. Examining the connection between ESCs’ immunomodulatory capabilities and tissue repair is crucial due to the anti-inflammatory and immunomodulatory qualities SCs possess (Liu et al., 2019). Diabetic mice have a delayed rise in macrophages and a longer inflammatory stage. To demonstrate their anti-inflammatory role in DFU healing, researchers found that the local proinflammatory reaction was dramatically decreased in the MSC group in comparison to the control group, and that CD45 expression was blocked. Diabetic mice might even recover more slowly because of aberrant chemokine release (Yang et al., 2020b). Increased production of CCL2 may restore normal skin wound healing in diabetic mice by mediating neovascularization and normalizing collagen deposition, contrary to the findings of previous research showing that CCL2 content in DW is lowered (Krzyszczyk et al., 2020). Both VEGF and transforming growth factor beta (TGF-β) were produced mostly by macrophages in the lesion, and DM may slow the proliferation of macrophages at the wounded location. Numerous reports have shown that MSCs may increase VEGF and TGF- β levels in DW locations. Yang et al. (2020b) showed that wound repair was greatly aided by an increase in angiogenesis following topical MSC administration and the expression of CCL2 was also significantly elevated. CCL2 receptors are expressed on macrophages (CCR2). Accordingly, it is reasonable to assume that CCL2 plays a crucial role in facilitating wound regeneration by reversing the decreased macrophage infiltration and increasing the levels of VEGF and TGF-β (Fujiwara and Kobayashi, 2005). TGF-β could control macrophage activity and proliferation by acting directly on Treg cells. The epidermal keratinocytes’ production of CCL2 is directly controlled by the transcription factor Nrf2 (Sierra-Filardi et al., 2014). Kato et al. (2014) found that BM-MSCs accelerated the healing of plantar skin lesions in diabetic mice through stimulating keratinocytes. Thus, MSCs may aid in the functional recovery of keratinocytes, causing a rise in the production of CCL2, which would revert the reduction in macrophage infiltration and aid in ulcer healing. EGF may stimulate keratinocyte growth, and CCL2 can control macrophages’ synthesis of it at the site of the injury. Reduction in EPCs, impairment in EPC function, and impaired recruitment to the wounded site have all been linked to hyperglycemia (Yang et al., 2020b). In diabetic mice, elevated CCL2 expression may promote the recruitment of EPCs to wound sites and hasten the development of neovascularization (Ishida et al., 2019). Increased production of proinflammatory cytokines such as interleukin-1, interferon-gamma, TNF-α, and interleukin-6 are hallmarks of diabetic chronic non-healing wounds (Shen et al., 2021). It has been hypothesised that impaired DW healing might result from a combination of decreased macrophage numbers and increased activation (Kong and Gao, 2017). Microglia/SCs applied topically have the potential to dampen inflammation by producing the anti-inflammatory cytokine IL-10. Wang et al. (2016) found that therapy with placenta-derived MSCs (PMSCs) plus IL-10 antibodies substantially slowed DW healing compared to therapy with PMSCs alone. In addition, PMSCs were shown to drastically lower local levels of IL-1, TNF-α, and IL-6 after being applied topically. In DB/DB mice, the onset of inflammation was noticeably delayed, and a substantial rise in pro-inflammatory cytokines (such as IL-1 and TNF-α) was still detectable even after the wounds healed. Strong suppression of lipopolysaccharide (LPS)-induced NF-kB activating in dermal fibroblasts by PMSCs was observed (Varela et al., 2019). It is postulated that PMSCs might control the inflammatory response in DFU recovery by addressing NF-kB, since NF-kB is crucial in regulating the production of the pro-inflammatory cytokines IL1, TNF- α, and IL-6. In addition, the MSC combination regimen was shown to have an immunomodulatory effect on injuries of DB/DB animals by drastically decreasing the levels of IL-1 and IL-6. Finally, the results show that MSCs aid in the healing of cutaneous wounds by reducing the release of pro-inflammatory cytokines and boosting the production of anti-inflammatory cytokines in the wound area (Shen et al., 2021). Macrophage phagocytosis is disrupted in diabetic wounds, and macrophage infiltration and inflammation are sluggish. M1 macrophages are activated by the presence of pro-inflammatory cytokines (IL-6, TNF- α, IFN-α). Anti-inflammatory cytokines such as IL-4, IL-10, and TGF- β may trigger the transition from M1 to M2 macrophages, which are characterized by the production of TGF- β and IL-10 (Krzyszczyk et al., 2020). Studies have demonstrated that M2 macrophages boost angiogenesis, reduce nerve injury, and suppress inflammation. Macrophages take on the characteristics of the surrounding inflammatory milieu in wounds. SCs applied topically to wounds have been shown to induce a switch in macrophages to an anti-inflammatory phenotype (M2) (Boodhoo et al., 2022). Reduced Treg levels cause prolonged re-epithelialization and reduced vascular maturation, two hallmarks of compromised healing (Sierra-Filardi et al., 2014; Liu et al., 2019; Olson et al., 2021). Patients with diabetic feet have been reported to see a dramatic improvement in the Treg/Th17 and Th2/Th1 cell ratios after receiving intramuscular injections of hUC-MSCs. There is a wide variety of macrophages, each with its own phenotype and set of specialized tasks that are controlled by its immediate microenvironment (Xia et al., 2021). There are two main subsets of macrophages: 1) Classically activated or M1 macrophages, that release pro-inflammatory cytokines including IL-1, IL-6, IL-12, IL-23, and TNF- α as a result of being polarised by LPS, either on their own or in combination with Th1 cytokines like IFN- γ and GM-CSF; and 2) Alternatively activated or M2 macrophages, that are immunoregulatory and anti-inflammatory. They are polarised by Th2 cytokines like IL-4 and IL-13 and secrete anti-inflammatory cytokines like IL-10 and TGF- β (Philippidis et al., 2004). Macrophages M1 and M2 have distinct roles and transcriptional patterns (Krzyszczyk et al., 2018). They may eradicate dangerous microorganisms and cure inflammation-related damage. M1/M2 macrophage polarization determines an organ’s fate during inflammation or injury. When infection or inflammation is powerful enough to affect an organ, macrophages release TNF-, IL-1, IL-12, and IL-23. Too much time in the M1 phase might injure tissues. M2 macrophages release IL-10 and TGF- β to decrease inflammation, help in tissue repair, remodeling, vasculogenesis, and maintain homeostasis. The first portion covers the origin, differentiation, activation, tissue distribution, plasticity and polarization, migration, antigen presentation, cytokine and chemokine production, metabolism, and microRNAs in macrophage polarization and function. Second, we discuss macrophage subsets’ beneficial and detrimental functions in normal and abnormal pregnancies, anti-microbial defense, anti-tumor immunity, metabolic disease and obesity, asthma and allergy, atherosclerosis, fibrosis, wound healing, and autoimmune (Loretelli et al., 2020).

### 4.2 Cellular mechanisms

#### 4.2.1 Transdifferentiation into epithelial cells, collagen deposition, *etc.*

Human umbilical cord-derived hUC-MSCs could actively transdifferentiate into epithelial cells and endothelial cells, which have a role in facilitating the healing of DFU, as well as stimulating angiogenesis *via* paracrine. It was thought that CD31^+^ cells would indicate transdifferentiated endothelial cells. It is hypothesised that hMSCs share traits with ADSCs which allow them to develop into endothelial cells and neovascularization (Caplan and Dennis, 2006). MAPK/ERK signalling route facilitates VEGF-induced BM-MSC to endothelial cell differentiation, whereas the PI3K signalling pathway regulates ADSC to endothelial cell differentiation (Yu et al., 2022b). Uncertainty exists about the signal route that hUC-MSCs use to differentiate into endothelial cells. MSCs may increase collagen production and encourage collagen formation in DFU (Lin et al., 2022). The production and distribution of collagen, which is largely found in the dermis of the skin and is released by fibroblasts, is intimately associated with the healing of skin injuries. DFU-derived fibroblasts have less proliferative capability and produce fewer growth factors (Zeidi et al., 2020). To examine the impact of co-culturing healthy fibroblasts and human umbilical cord blood-derived MSCs (hUCB-MSCs) with diabetic fibroblasts on cell proliferation and collagen formation, both types of fibroblasts were employed (Al Sadoun, 2022). According to the findings, hUBC-MSCs did not produce a higher level of ECM component secretion compared to fibroblasts. However, hUBC-MSCs significantly increased the production of ECM (including collagen) in diabetic fibroblasts in comparison to fibroblasts. Consequently, MSCs might encourage granulation tissue development in DFUs by fostering fibroblast proliferation and functional recovery, which in turn prompts fibroblasts to release additional ECM and growth regulators and eventually propagates tissue repair (Shi et al., 2020). The SC healing of a diabetic foot is a multi-step procedure that includes several interrelated systems that work together to enhance damage repair in the end. For instance, macrophage M2 polarisation speeds up the healing of peripheral nerves (Yang et al., 2020a).

The aforementioned potential benefits of MSCs for improving diabetic wound healing have led to the application of MSC therapy at clinical levels and the designation of several clinical studies. Table 1 lists randomized controlled trials that have inquired about the effects of MSC therapy in patients with DFU. As can be seen, these studies mostly confirm the positive role of MSCs in enhancing the healing process and improving clinical parameters.

**TABLE 1:** Randomized controlled trials investigating the effects of mesenchymal stem cell therapy in patients with diabetic foot ulcers.

First author	Year	Country	Number of patients with DFU	Type of MSC	Administration route	Follow-up duration	Outcomes
Debin et al. (2008)	2008	China	50	BM-MSC	Intramuscular and subcutaneous	12 weeks	BM-MSC improved ischemic symptoms, healing process, ABI, neovascularization, and limb amputation rate
Dash et al. (2009)	2009	India	6	BM-MSC	Intramuscular	12 weeks	BM-MSCs enhanced the healing process and clinical parameters
Lu et al. (2011)	2011	China	41	BM-MSC	Intramuscular	24 weeks	BM-MSC ameliorated wound healing, limb perfusion, ABI, transcutaneous oxygen pressure, and magnetic resonance angiography findings
Jain et al. (2011)	2011	India	48	BM-MSC	Injection and spray	3 months	BM-MSC improved wound healing
Kirana et al. (2012)	2012	Germany	24	BM-MSC	Injection and intra-arterial	45 weeks	BM-MSC enhanced wound healing and ABI
Qin et al. (2016)	2016	China	53	hUC-MSC	Intramuscular and intra-arterial	3 months	hUC-MSC improved ABI, skin temperature, and claudication
Albehairy et al. (2018)	2016	Egypt	18	BM-MSC	Intramuscular	12 weeks	Significant reduction in ulcer surface area
Moon et al. (2019)	2019	South Korea	59	ASC	Dressing with ASC sheet	12 weeks	ASC sheet dressings were associated with higher rates of complete wound closure
Lu et al. (2019)	2019	China	41	BM-MSC	Intramuscular	3 years	Significant improvement in wound healing and recurrence rate
Lonardi et al. (2019)	2019	Italy	114	ASC	Intramuscular	6 months	ASC improved healing rate following minor amputations of non-healing DFU
Arango-Rodríguez et al. (2022), Askø Andersen et al. (2022)	2022	Colombia	28	BM-MSC	Intradermal	The wound healing process was monitored until the complete closure of the ulcers	Higher percentages of wound closure, improved skin regeneration, and an enhanced ulcer-free survival

Abbreviatuions: DFU, diabetic foot ulcer; BM-MSC, bone marrow derived mesenchymal stem cell; hUC-MSC, human umbilical cord mesenchymal stem cell; ABI, ankle-brachial index; PB-MSC, peripheral blood derived mesenchymal stem cell; ASC, adipose-derived stem cells.

## 5 Conclusion

To summarize, MSC therapy is an advanced technology with great potential in the management of non-healing DFU. A vast body of evidence confirms the involvement of MSCs in all phases of cutaneous wound healing including homeostasis, inflammatory, proliferative, and remodeling phases. MSCs as pluripotent mesoderm-derived cells can differentiate into various types of cells. This property enables them to act effectively in the field of tissue repair by providing the injured sites with their demolished native components. Additionally, MSCs ameliorate neovascularization in the DFU site by generating angiogenic factors above all VEGF. MSCs can modulate the immune system by affecting the immune responses of T cells and macrophages significantly as well as reducing the local levels of pro-inflammatory cytokines IL-1, TNF-α, and IL-6, among others. In animal models and clinical trials, the administration of MSCs for non-healing DFUs has been associated with promising results. Thus, this technique provides a new horizon for improving the clinical outcomes of diabetic patients suffering from DFU. Nevertheless, future studies are required to address some issues in this field including investigating the possibility of unrestricted cell differentiation and the development of tumors after receiving MSC therapy; (Han et al., 2022); introducing methods to obtain high levels of MSCs with the utmost purity; and (Mahant et al., 2021) optimizing methods to evaluate the long-term efficacy of MSCs.
